# Successful treatment of severe immune checkpoint inhibitor associated autoimmune hepatitis with basiliximab: a case report

**DOI:** 10.3389/fimmu.2023.1156746

**Published:** 2023-05-30

**Authors:** Maiah Zarrabi, Camille Hamilton, Samuel W. French, Noah Federman, Theodore S. Nowicki

**Affiliations:** ^1^ Department of Pediatrics, University of California, Los Angeles Mattel Children’s Hospital, Los Angeles, CA, United States; ^2^ Division of Pediatric Hematology Oncology, Department of Pediatrics, University of California, Los Angeles Mattel Children’s Hospital, Los Angeles, CA, United States; ^3^ Department of Pathology, David Geffen School of Medicine at University of California, Los Angeles, Los Angeles, CA, United States; ^4^ Department of Microbiology, Immunology, and Molecular Genetics (MIMG), University of California, Los Angeles, Los Angeles, CA, United States

**Keywords:** immune evasion, immunohistochemistry, immunotherapy, programmed cell death, case report

## Abstract

Immune checkpoint inhibitors (ICIs) targeting programmed cell death-1 (PD-1) and its corresponding ligand PD-L1 are being increasingly used for a wide variety of cancers, including refractory sarcomas. Autoimmune hepatitis is a known side effect of ICIs, and is typically managed with broad, non-specific immunosuppression. Here, we report a case of severe autoimmune hepatitis occurring after anti-PD-1 therapy with nivolumab in a patient with osteosarcoma. Following prolonged unsuccessful treatment with intravenous immunoglobulin, steroids, everolimus, tacrolimus, mycophenolate, and anti-thymoglobulin, the patient was eventually treated with the anti-CD25 monoclonal antibody basiliximab. This resulted in prompt, sustained resolution of her hepatitis without significant side effects. Our case demonstrates that basiliximab may be an effective therapy for steroid-refractory severe ICI-associated hepatitis.

## 1 Introduction

Immune checkpoint inhibitors (ICIs) targeting programmed cell death-1 (PD-1) and its corresponding ligand PD-L1 are being increasingly used for a wide variety of cancers, including refractory sarcomas (1). Autoimmune hepatitis is a known side effect of ICIs, typically occurring 8-12 weeks after initiation. It is seen in 5-15% of patients using a single agent ICI, but only 1-3% experience grade 3-4 hepatotoxicity (2). First-line therapy remains discontinuation of the ICI and initiation of steroids, with some literature supporting T-cell-specific immunomodulators such as mycophenolate, tacrolimus, or anti-thymoglobulin (ATG) as second line agents (2, 3). No current recommendations exist for patients with ICI induced autoimmune hepatitis resistant to these first- and second-line agents. Here we describe a patient with an asymptomatic, isolated rise in liver function tests in the setting of nivolumab therapy, and our experience utilizing basiliximab, an anti-CD25 monoclonal antibody, to successfully manage her severe, steroid-refractory autoimmune hepatitis.

## 2 Case description

A 22-year-old female with recurrent metastatic osteosarcoma of her right proximal tibia began treatment with the anti-PD-1 monoclonal antibody nivolumab for refractory disease.

**Figure 1:**
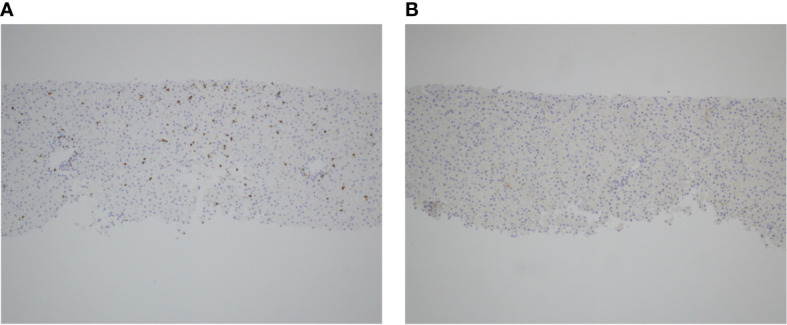
T-cell infiltration in the setting of anti-PD-1 induced autoimmune hepatitis. Liver biopsy obtained during acute hepatitis demonstrates significant infiltration of CD3+ T-cells **(A)** and scattered CD25+ T-cells **(B)**, as shown by immunohistochemistry staining for both markers, respectively.

**Figure 2:**
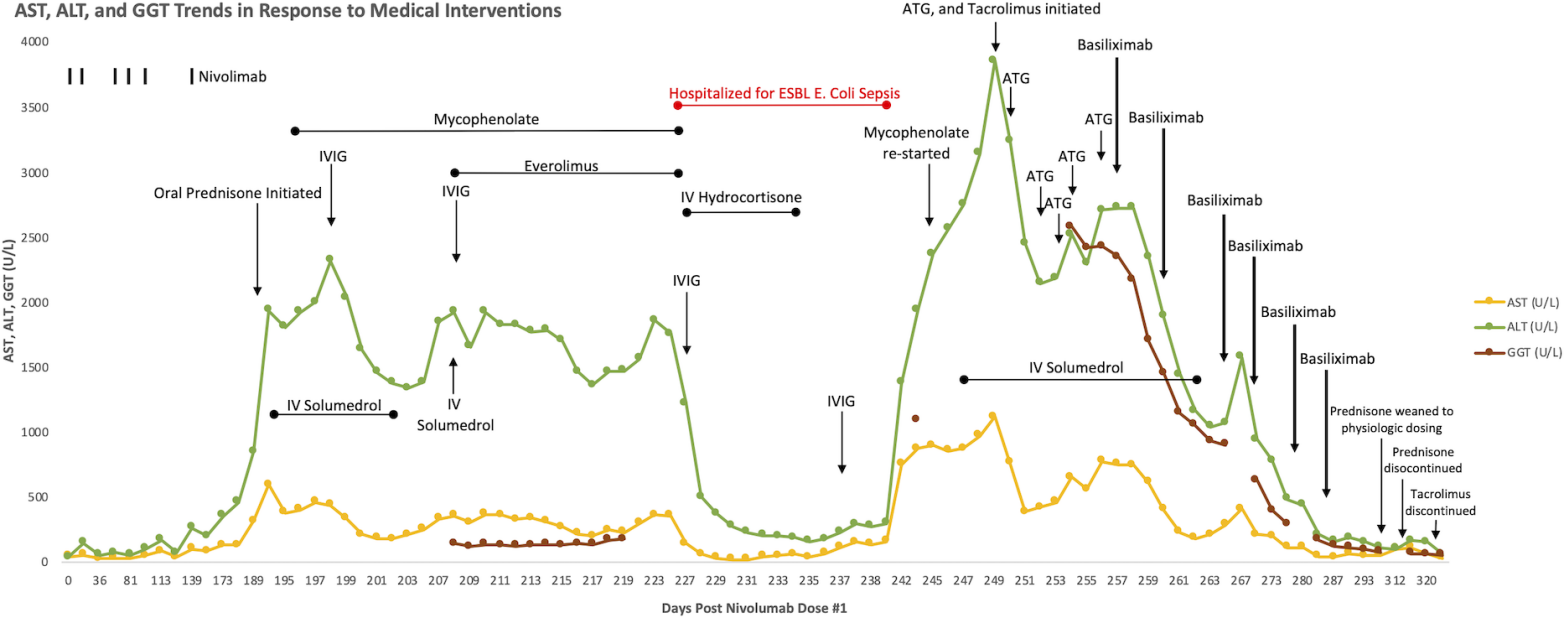
Graphical display of rising AST, ALT, and GGT over time in response to nivolumab therapy, refractory to steroids, mycophenolate, everolimus, IVIG, tacrolimus, rabbit ATG, and IVIG. There is subsequent down trending and persistent suppression of AST, ALT, and GGT following initiation of basiliximab, despite discontinuation of steroids and tacrolimus.

After five months of therapy with a total of six doses of nivolumab (480 milligrams/dose), the patient developed new onset hepatitis indicated by isolated rising liver function tests (LFTs), identified on routine blood work. She reported no new symptoms and was not jaundiced on exam. Nivolumab was discontinued at this time. LFTs continued to rise despite discontinuation of nivolumab, with alanine aminotransferase (ALT) elevated to 847 U/L (reference range 8-70 U/L) and aspartate aminotransferase (AST) elevated to 314 U/L (reference range 13-62 U/L), both of which had been previously within normal limits at her baseline. She was initially treated with oral prednisone, but her LFTs continued to rise. She was admitted to an academic medical center for an intravenous (IV) steroid pulse in conjunction with initiation of mycophenolate and intravenous immunoglobulin (IVIG). An infectious workup was negative for any infectious etiology explaining her hepatitis, and a liver biopsy showed scattered apoptotic hepatocytes with lobular T-cell infiltrates (CD3+, CD8+, CD25+) but no steatosis or fibrosis (**Figure 1**). Given the continued rise in her LFTs despite these interventions, further treatments were initiated including everolimus, rabbit ATG, and tacrolimus. None of these interventions were successful in persistently suppressing her LFTs, which peaked at greater than 20 times the upper limit of normal (ALT peaked at 3856 U/L and AST peaked at 1117 U/L on day 249 status post the first nivolumab dose, and γ-glutamyltransferase (GGT) peaked at 2577 U/L (reference range 7-68 U/L) on day 254 status post the first nivolumab dose) (**Figure 2**), reflecting a grade 4 hepatitis (2).

While immunosuppressed secondary to treatment, she suffered extended spectrum beta-lactamase (ESBL) Escherichia coli sepsis leading to acute hypoxic respiratory failure requiring intubation and vasopressors. The source of her sepsis was thought to be either a central line-associated bloodstream infection, or a pneumonia with subsequent bacteremia, as her bronchioalveolar lavage sample was positive for the same bacteria as was in her blood. She received stress-dose steroids during this time and her LFTs down trended, however this response was not sustained. Given her persistently rising LFTs with minimal response to the aforementioned therapies, basiliximab, given its specificity against activated T-cells and its established use in preventing acute rejection in solid organ transplants, was initiated while continuing tacrolimus, mycophenolate, and steroids. She received a total of six doses of basiliximab (20 milligrams/dose), the initial two during the first week for a loading dose effect (given on day 1 and day 4), then weekly thereafter for an additional four weeks. Her steroids were weaned to physiologic doses and subsequently discontinued, and tacrolimus and mycophenolate have been discontinued, with normalization of her LFTs and no major side effects secondary to basiliximab.

## 3 Discussion

PD-1 and its ligand PD-L1 are important immune checkpoints by which cancer cells evade the host immune response (4). PD-1 regulates the effector phase of CD8+ T-cells after chronic antigen exposure. PD-L1 is a cell-surface protein induced in many malignancies in response to local inflammatory stimuli, such as interferons. By acting on PD-1, PD-L1 suppresses anti-tumor T-cell responses (4), a phenomenon known as immune escape. This new paradigm led to the clinical development of antibodies blocking PD-1 or PD-L1, which have shown durable clinical responses in a variety of malignancies (1). However, ICIs can disrupt immune homeostasis and lead to autoimmune side effects. This phenomenon is multifactorial and involves both T-cell activation and proliferation in response to host antigens, as well as disruption of regulatory T-cell function. A histology review in 2018 observed that immunohistochemical stains of ICI-associated liver injury were distinguished by infiltration of CD3+ and CD8+ T-cells, as compared to stains of classic autoimmune hepatitis and other drug-induced liver injury which are marked by relatively higher quantities of CD4+ T cells and CD20+ B-cells (5).

We describe herein a case of steroid-refractory severe ICI-associated autoimmune hepatitis caused by nivolumab, which was refractory to multiple nonspecific immunosuppressive interventions, including systemic steroids, mycophenolate, IVIG, everolimus, tacrolimus, and rabbit ATG. The hepatitis eventually responded favorably to basiliximab, following continued elevation in LFTs despite all prior interventions. Basiliximab is an anti-CD25 monoclonal antibody that blocks the interaction of interleukin-2 with its receptor IL-2Rα, therefore specifically depleting activated effector T-cells (6). Basiliximab is used in practice as induction therapy to prevent acute rejection in solid organ transplants (6) and is also utilized in steroid-refractory acute graft-versus-host disease following allogenic hematopoietic stem cell transplantation (7). To our knowledge, basiliximab has not previously been used to treat ICI-associated autoimmune hepatitis.

Although our patient’s LFTs demonstrated a modest response initially to a combination of high dose steroids, mycophenolate, and everolimus in conjunction with IVIG, she was left profoundly immunocompromised and required intensive care unit admission for septic shock. Significant risk of opportunistic infections in the setting of such broad and non-specific immunosuppression is an important consideration when managing patients with immune-related adverse events (irAEs). A 2016 study showed that in 740 patients treated with ICIs for metastatic melanoma, there was an overall 7.3% incidence of serious infection, including that from bacterial, viral, fungal, and parasitic sources. The use of corticosteroids to treat irAEs in these patients was associated with the greatest increase in risk for serious infection (8). After recovering from sepsis, our patient’s hepatitis persisted and so tacrolimus, previously avoided due to its longer onset of action, was initiated along with ATG, which has been reported to resolve severe ICI-associated hepatitis (3), although neither led to any significant decrease in the patient’s LFTs. It was not until basiliximab was initiated that her LFTs displayed a consistent downward trend, to a point where it was feasible to discontinue her steroids and other immunosuppressive medications. Basiliximab’s effectiveness in this setting is likely attributed to its specific binding to IL-2Rα which is found on activated T-cells, with resultant inhibition of the activated T-cell proliferation, clearance of activated T-cells, and suppression of pro-inflammatory cytokine production, which were contributing to hepatocyte destruction in our patient. While synergistic effects of the basiliximab with other T-cell-specific agents (e.g tacrolimus, mycophenolate) is plausible, we did not observe consistent, sustained improvement until basiliximab’s addition to the immunosuppressive regimen, highlighting its essential role in reversal of the patient’s ICI-induced autoimmune hepatitis.

In conclusion, basiliximab successfully treated our patient’s ICI-induced autoimmune hepatitis without major side effects. This was in the setting of our prior treatment strategy of using a wide variety of nonspecific immunosuppressive interventions failing to achieve sufficient mitigation of her hepatitis. While ICIs are effective treatments for a variety of advanced cancer subtypes, clinicians require additional means of managing their autoimmune toxicities (9). Our case demonstrates that basiliximab may be an effective therapy for steroid-resistant severe ICI-associated autoimmune hepatitis. Our success highlights the need for further studies, including randomized controlled trials studying safety and efficacy, to determine the best modality of treatment for patients with ICI-induced adverse effects.

## 4 Patient perspective

Successful treatment of her hepatitis allowed our patient to be safely discharged from the hospital, and she continues supportive care for her sarcoma on an outpatient basis. One year following her admission, her LFTs remain normal without immunosuppressive medication. She enthusiastically consented to having her case shared with the hope of helping future patients.

## Data availability statement

The original contributions presented in the study are included in the article/supplementary material. Further inquiries can be directed to the corresponding author.

## Ethics statement

Ethical review and approval was not required for the study on human participants in accordance with the local legislation and institutional requirements. The patients/participants provided their written informed consent to participate in this study. Written informed consent was obtained from the individual(s) for the publication of any potentially identifiable images or data included in this article.

## Author contributions

MZ and CH cared for the patient, drafted the initial manuscript, and reviewed and revised the manuscript. SF was involved with the diagnostic workup of the patient and reviewed and revised the manuscript. NF and TN cared for the patient, were involved with the diagnostic workup and therapeutic plan for the patient, and critically reviewed and revised the manuscript. All authors contributed to the article and approved the submitted version.
